# A meta-synthesis of qualitative research on negative body image among female breast cancer patients: Perceptions, stigma, and identity negotiation

**DOI:** 10.1097/MD.0000000000049592

**Published:** 2026-07-03

**Authors:** Long Yao, Li Ling, Tuo Xuemei, Yu Dan, Huang Yali

**Affiliations:** a Chongqing Liangjiang New Area Traditional Chinese Medicine Hospital, Chongqing, China; b Department of Rehabilitation, Chongqing Liangjiang New Area Traditional Chinese Medicine Hospital, Chongqing, China; c Department of Nursing, Chongqing Liangjiang New Area Traditional Chinese Medicine Hospital, Chongqing, China; d Department of Neurology, Chongqing Liangjiang New Area Traditional Chinese Medicine Hospital, Chongqing, China.

**Keywords:** body image, breast cancer, meta-synthesis, psychosocial intervention, qualitative research, stigma, survivorship

## Abstract

**Background:**

Negative body image is a consistently reported psychosocial concern among female breast cancer (BC) survivors, yet the full scope of their lived experiences across different cultural and clinical contexts has not been comprehensively synthesized. A deeper understanding of how women perceive and navigate body image disturbances following diagnosis and treatment is essential for developing responsive supportive care.

**Methods:**

Ten databases were systematically searched for relevant publications published up to June 2025. Eligible studies explored the body image experiences of adult female patients diagnosed with BC through qualitative research. The Australian Joanna Briggs Institute Critical Appraisal Checklist for Qualitative Research was used for quality evaluation, and the results were integrated using thematic analysis.

**Results:**

Nineteen studies were included. From these studies, 40 distinct findings (e.g., significant statements, themes, or concepts reported by the original study authors) were extracted and subsequently synthesized into 12 subthemes and 4 main themes: physical changes and how they affect self-image, dealing with others’ judgments and social challenges, rebuilding a positive self-image, and body image problems and intimate relationships.

**Conclusion:**

Individuals who have survived BC often experience significant disturbances in their body image. These disturbances are characterized by disruption of identity, social stigma, relational strain, and efforts to achieve self-acceptance and create a meaningful existence. These findings emphasize the necessity for early psychosocial assessment, targeted interventions such as mirror exposure therapy, and the development of multilevel support systems to enhance body image adjustment and overall quality of life in survivorship care.

## 1. Introduction

Breast cancer (BC) is one of the most prevalent malignancies affecting females worldwide.^[1]^ In 2022 alone, it accounted for an estimated 2.3 million new diagnoses and affected the lives of 7.8 million females living with the disease.^[2]^ According to 2025 projections from the International Agency for Research on Cancer, approximately 1 in 20 females globally will be diagnosed with BC in their lifetime, and 1 in 70 will die from the disease.^[3]^ Approximately 99% of BCs occur in females, and 0.5% to 1% of BCs occur in males.^[2]^ With the development of diagnostic and therapeutic techniques, the 5-year survival rate for BC has reached 90%.^[4]^ Mastectomy remains a common and effective prophylactic and therapeutic intervention; however, it frequently induces visible bodily changes such as breast loss, alopecia, and edema.^[5]^ Although breast reconstructive surgery can mitigate some negative postmastectomy experiences for certain female patients,^[6,7]^ the resultant physical scarring and bodily changes can become a central, disruptive focus of self-identity. For many females, this leads to feelings of being “mutilated,” unfeminine, and disembodied.^[8,9]^ Studies have consistently shown that the permanent changes to key parts of the body disturb body perception, body identity, and body shape cognition in patients with BC during the process of accepting physical changes.^[8–11]^

Body image is a multidimensional construct encompassing self-representations, emotions, and behaviors related to physical appearance and bodily function.^[12]^ A distorted body image is a frequently reported feature of the BC experience, particularly as female patients confront the dissonance between their pre- and post-cancer bodies and grapple with aspects that may never fully recover.^[13]^ When patients perceive the difference between their actual appearance and the ideal state after a mastectomy, body image disturbance reaches its peak, causing depression, social withdrawal, and functional impairment.

In contemporary society, female patients are consistently confronted with idealized, often unattainable, beauty standards, which many female BC patients internalize as benchmarks for self-evaluation. However, the posttreatment body image often no longer aligns with female patients’ internalized self-image or mainstream social norms, causing significant psychological distress, which often persists for a long period.^[14]^ Given the negative impact of mastectomy on mental health and overall well-being, it is crucial to understand the factors that cause and perpetuate body image distress.

Qualitative research plays a key role in foregrounding the lived experiences and personal significance of these physical changes. An increasing number of qualitative research documents explore different cultural contexts and provide rich, nuanced insights directly from the perspective of BC survivors. To consolidate this knowledge and inform future care, this study employed a meta-synthesis to integrate findings from qualitative studies exploring the negative body image experiences of female patients diagnosed with and treated for BC. This study aimed to provide a robust evidence base for the development of effective supportive interventions, which may be delivered by nurses or other healthcare professionals (e.g., allied health professionals and psychologists).

## 2. Methods

### 2.1. Design

Meta-synthesis is a rigorous research method designed to analyze and synthesize findings from existing qualitative studies. Meta-synthesis aims to construct higher-order interpretations by identifying overarching themes.^[15]^ Guided by Thomas and Harden’s thematic analysis method,^[16]^ this study extracts the key contents of the literature and analyzes and synthesizes their significance and essence. The data extraction and integration procedure consists of 3 stages: coding textual findings, developing descriptive themes, and constructing analytical themes.

### 2.2. Search strategy

We conducted a literature search for qualitative studies on body image in BC patients using the following databases: PubMed, Wiley, APA PsycINFO (EBSCOhost), Cumulative Index to Nursing and Allied Health Literature (EBSCOhost), Web of Science, Embase, China National Knowledge Infrastructure, VIP, Wanfang Data, and SinoMed. The search time limit for the self-built library was June 2025. We employed Medical Subject Headings and online synonyms to identify keywords and their derivatives and used a combination of subject terms and free words as search strategies. We also conducted additional retrospective searches of the reference lists of included studies. The detailed search strategies for each database are provided in Appendix 1, Supplemental Digital Content 1.

### 2.3. Literature inclusion and exclusion criteria

Inclusion criteria were as follows: participants, female BC patients aged ≥18 years; phenomenon of interest, BC patients’ perceptions of body image changes; research context, the period following BC surgery or chemotherapy; and research design, qualitative studies, including but not limited to phenomenology, descriptive qualitative research, grounded theory, and ethnography.

The exclusion criteria were as follows: duplicate publications; full-text unavailability; studies not published in English or Chinese; mixed-methods studies in which qualitative findings could not be clearly separated and analyzed; and non-qualitative studies, including quantitative studies, randomized controlled trials, literature reviews, systematic reviews, conference abstracts, case reports, and theoretical papers.

### 2.4. Literature screening and data extraction

Two researchers (LY and TXM) independently retrieved and screened the studies according to the inclusion and exclusion criteria, extracted data, and cross-checked the findings. EndNote X9 software (Clarivate Analytics) was used to eliminate duplicate documents. We reviewed the titles and abstracts to exclude records that did not meet the predefined inclusion and exclusion criteria. Full-text articles were carefully assessed. Disagreements in the research process were resolved through arbitration by a third researcher (HYL) or through team discussion.

We developed a data extraction proforma encompassing the first author, country, publication year, research purpose, aims, sample size, analysis methods, and the main results. Data extraction was conducted by LY, whereas LL conducted a subsequent review to ensure the veracity of the information. Discrepancies were resolved through discussion until consensus was reached. The extracted data are listed in Table 1.

**Table 1:** Characteristics of the included studies (n = 19).

Year	Author	Country	Methodology	Data collection	Sample		Aim	Results
					N	Age		
2024	González-Fernández et al^[23]^	Spain	Descriptive qualitative study	Unstructured interview, semi-structured interview, interviewer’s field notes, and personal letters	20	37–82 (average 56.15)	Effect of lymphedema of breast cancer on body image	From cancer to lymphedema, another disease
								Potential for transition and transformation towards a new way of life
2024	Davis et al^[27]^	United States	Descriptive qualitative study	Semi-structured interview, online interview	10	48–80 (average 64)	Body image concerns of breast cancer survivors	Age at diagnosis
								Intimate partnership
								Preparedness for physical changes
								Mental fluidity
2024	Cernikova et al^[14]^	Czech Republic	DIPEx methodology	Deep interview, semi-structured interview	53	28–76 (average 48.61)	Body image changes of female patients with breast cancer in the whole disease trajectory	Pre-diagnosis
								Diagnosis (emotions uncertainties, coping mechanisms, and strategies)
								Treatment (psychological impact and identity, adaptive coping, and self-care)
								Posttreatment (psychological impact of body image changes, adoption of breast substitutes)
								Survivorship (importance of physical appearance and social perception, intimate relationships and self-image, permanent reminders and body image issues, self‑esteem and acceptance)
2023	Hasan et al^[28]^	Syria	Phenomenological qualitative study	Semi-structured deep interview	10	28–48	Effect of mastectomy on psychological, emotional, and social aspects of young female breast cancer patients in Syria	Psychological and emotional well-being
								Body image and breast reconstruction
								Social and interpersonal factors
								Coping mechanisms with mastectomy effects
								Physical health and functioning
2022	Sebri et al^[18]^	Italy	Descriptive qualitative study	Online questionnaire	20	38–69 (average 50.6)	Cognitive and emotional changes of breast cancer survivors on body	Relationship with the body
								Emotions
								Thoughts
2022	Brunet et al^[20]^	Canada	Phenomenological qualitative study	Semi-structured interview	27	25–81 (average 56.23)	The meaning of body image of female patients with breast cancer and how to treat the influence of breast cancer experience on body image	Treatment-related events can undermine or support body image
								Psychosocial factors can undermine or support body image
								Sociocultural factors can undermine body image
								Repertoire of strategies to manage body image
								Passage of time
								Consequences of body image
2019	Peerawong et al^[33]^	Thailand	Descriptive qualitative study	Semi-structured interview, focus group	25	18–50	Changes of body image of female breast cancer patients after diagnosis and operation	The moment of diagnosis and changed self
								Transition and recovery
								Normalization
2019	Herring et al^[21]^	United Kingdom	Descriptive qualitative study	Open-ended question	128	31–74 (average 51.6)	Female breast cancer patients’ initial experience of their appearance after surgery	Preparedness and support
								First thoughts and emotions
								Loss and grief
								The reaction of others
2019	Alhusban et al^[30]^	Jordan	Phenomenological qualitative study	Semi-structured interview	20	/	Perception of body image changes in patients with breast cancer	Broken things cannot be repaired
2019	Liu and Chen^[25]^	China	Phenomenological qualitative study	Semi-structured interview	9	48–72	Changes of body image, coping style, and demand of female patients with breast cancer at different stages of illness	Changes in body image
								Changes in coping
2018	Chuang et al^[34]^	Taiwan, China	Phenomenological qualitative study	Semi-structured interview	8	41–59	Body perception of female breast cancer patients after mastectomy	Restoration of the body image
								Abandonment of objectification
								Redefinition of self
2017	Grogan and Mechan^[22]^	United Kingdom	Descriptive qualitative study	Online questionnaire	49	29–53 (average 39)	Body image experience of young women after mastectomy	Downplaying aesthetics relative to surviving cancer
								Body confidence
								Changed identity
								Treatment effects
2017	Barthakur et al^[32]^	India	Descriptive qualitative study	Semi-structured deep interview	15	45–72	Body image and sex-related problems of breast cancer patients after diagnosis and treatment	Identity: womanhood, motherhood, and attractiveness
								Impact of surgery
								Hair loss
								Clothes
								Uncomfortable situations
								Sexuality
2016	Kocan and Gursoy^[31]^	Turkey	Descriptive qualitative study	Semi-structured interview	20	32–58 (average 45.9)	How does mastectomy affect the body image of women with breast cancer?	Meaning of the breast
								Mastectomy and me
								My body image and body image changes
								Social life
2016	Buki et al^[35]^	United States	Phenomenological qualitative study	Focus group, individual interview	27	35–68	Views of breast cancer survivors on body image	Perceptions of loss and reconstruction
								Process of achieving body image acceptance
2013	Gaines^[19]^	United States	Phenomenological qualitative research	Deep interview	12	35–63	Experience of body image after receiving breast cancer treatment	Feelings of shame and embarrassment related to bodily changes during and after treatment
								Breasts and hair as symbols of femininity and attractiveness
								Changes in intimate relationships
								Strategies for coping with breast cancer treatment and side effects, changes to body, and relationships
								Learning to accept a new normal
								Gratitude and meaning making
								The invisible scars
2013	Brunet et al^[29]^	Canada	Interpretative phenomenological analysis	Semi-structured interview	11	47–70	Experience of your body after receiving breast cancer treatment	Changing visibly and invisibly
								Experiencing intense thoughts and emotions
								The meaning of the body: a vehicle of health, well-being, and social expression
								Managing and dealing with physical changes
2013	Huang and Bao^[24]^	China	Descriptive qualitative study	Deep interview, field survey	14	31–74	Changes of body image of breast cancer patients under the framework of “disability”	Bodies branded with the “breast cancer” label
								The emergence of an “imperfect” body image and coping strategies
								The disruption and reconstruction of female body image
								Challenges and management in intimate relationships
								Crises and management of self and social identity
2001	Robson^[26]^	Canada	Interpretative phenomenological analysis	Deep interview	8	<50	Unique views on physical changes after breast cancer surgery	Body image as a personal perspective
								The body as a physical experience incorporates the body as an object and functioning instrument
								The public body includes the visible and observed body
								The private body as an expression of self refers to issues related to feminine identity
								Intimacy and sexuality; and reintegrating body image into the self

### 2.5. Appraisal of methodological quality

Methodological evaluation of the included literature was conducted using the JBI Critical Appraisal Tool (Joanna Briggs Institute, Faculty of Health and Medical Sciences, University of Adelaide)^[17]^ by 2 researchers (LY and YD). The evaluation encompassed a total of 10 aspects, each with 4 answers (yes, no, unclear, and not applicable). Any disagreements were resolved through arbitration by a third researcher (HYL) or through team discussion. The methodological quality assessment results of the included studies are presented in Table 2.

**Table 2:** Methodological quality assessment results of included studies (n = 19).

Year	Author	①	②	③	④	⑤	⑥	⑦	⑧	⑨	⑩	Level
2024	González-Fernández et al^[23]^	+	+	+	+	+	−	−	+	+	+	B
2024	Davis et al^[27]^	?	+	+	+	+	−	−	+	+	+	B
2024	Cernikova et al^[14]^	?	+	+	+	+	−	−	+	+	+	B
2023	Hasan et al^[28]^	?	+	+	+	+	+	−	+	+	+	B
2022	Sebri et al^[18]^	?	+	+	+	+	+	−	+	+	+	B
2022	Brunet et al^[20]^	?	+	+	+	+	−	+	+	+	+	B
2019	Peerawong et al^[33]^	?	+	+	+	+	−	−	+	+	+	B
2019	Herring et al^[21]^	+	+	+	+	+	+	−	+	+	+	B
2019	Alhusban et al^[30]^	+	+	+	+	+	−	−	+	+	+	B
2019	Liu and Chen^[25]^	?	+	+	+	+	−	−	+	+	+	B
2018	Chuang et al^[34]^	+	+	+	+	+	−	−	+	+	+	B
2017	Grogan and Mechan^[22]^	?	+	+	+	+	−	−	+	+	+	B
2017	Barthakur et al^[32]^	+	+	+	+	+	+	−	+	+	+	B
2016	Kocan and Gursoy^[31]^	?	+	+	+	+	−	−	+	+	+	B
2016	Buki et al^[35]^	?	+	+	+	+	−	−	+	+	+	B
2013	Gaines^[19]^	+	+	+	+	+	−	−	+	+	+	B
2013	Brunet et al^[29]^	+	+	+	+	+	−	−	+	+	+	B
2013	Huang and Bao^[24]^	?	+	+	+	+	+	−	+	?	+	B
2001	Robson^[26]^	+	+	+	+	+	+	+	+	+	+	A

Yes = “+”; No = “−”; Unclear = “?”.

Q1: Is there congruity between the stated philosophical perspective and the research methodology?

Q2: Is there congruity between the research methodology and the research question or objectives?

Q3: Is there congruity between the research methodology and the methods used to collect data?

Q4: Is there congruity between the research methodology and the representation and analysis of data?

Q5: Is there congruity between the research methodology and the interpretation of results?

Q6: Is there a statement locating the researcher culturally or theoretically?

Q7: Is the influence of the researcher on the research, and vice-versa, addressed?

Q8: Are participants, and their voices, adequately represented?

Q9: Is the research ethical according to current criteria or, for recent studies, is there evidence of ethical approval by an appropriate body?

Q10: Do the conclusions drawn in the research report flow from the analysis or interpretation of the data?

Adapted from JBI Critical Appraisal Tool (2015), https://jbi.global/critical-appraisal-tools.

### 2.6. Data synthesis

The meta-synthesis was guided by Thomas and Harden’s thematic analysis method.^[16]^ Word and Excel were used to facilitate data organization. Two researchers (LY and TXM) independently read the results extracted from the studies and quotations from the participants and coded them accordingly. The corresponding author (HYL) checked all original data and codes, and if there was any opinion, a group discussion was conducted to determine the coding situation. After coding all studies, LY and LL developed the themes. Second, LY constantly summed up and summarized similar and different coding situations to determine the themes and subthemes. Finally, LY read the initial data again to ensure that no other new codes or topics appeared, and the corresponding author checked all confirmed topics again.

### 2.7. Rigor, trustworthiness, and reflexivity

Team members have backgrounds in nursing and specialized training in evidence-based medicine. All included studies were independently screened and evaluated by 2 researchers. Thematic analysis was employed to systematically identify and interpret the data, facilitating a deeper understanding of the body image experiences of BC patients. To enhance credibility, quotations from representative participants were extracted to substantiate the developed themes. During the data analysis process, the researchers meticulously adhered to the original expressions of the participants, deliberately eschewing the imposition of personal interpretations.

### 2.8. Ethics statement

Meta-synthesis is a secondary analysis that does not involve the collection of new primary data from human participants. This study was exempt from formal ethical review and approval by an institutional review board.

## 3. Results

### 3.1. Basic characteristics of the study

**Figure 1.**
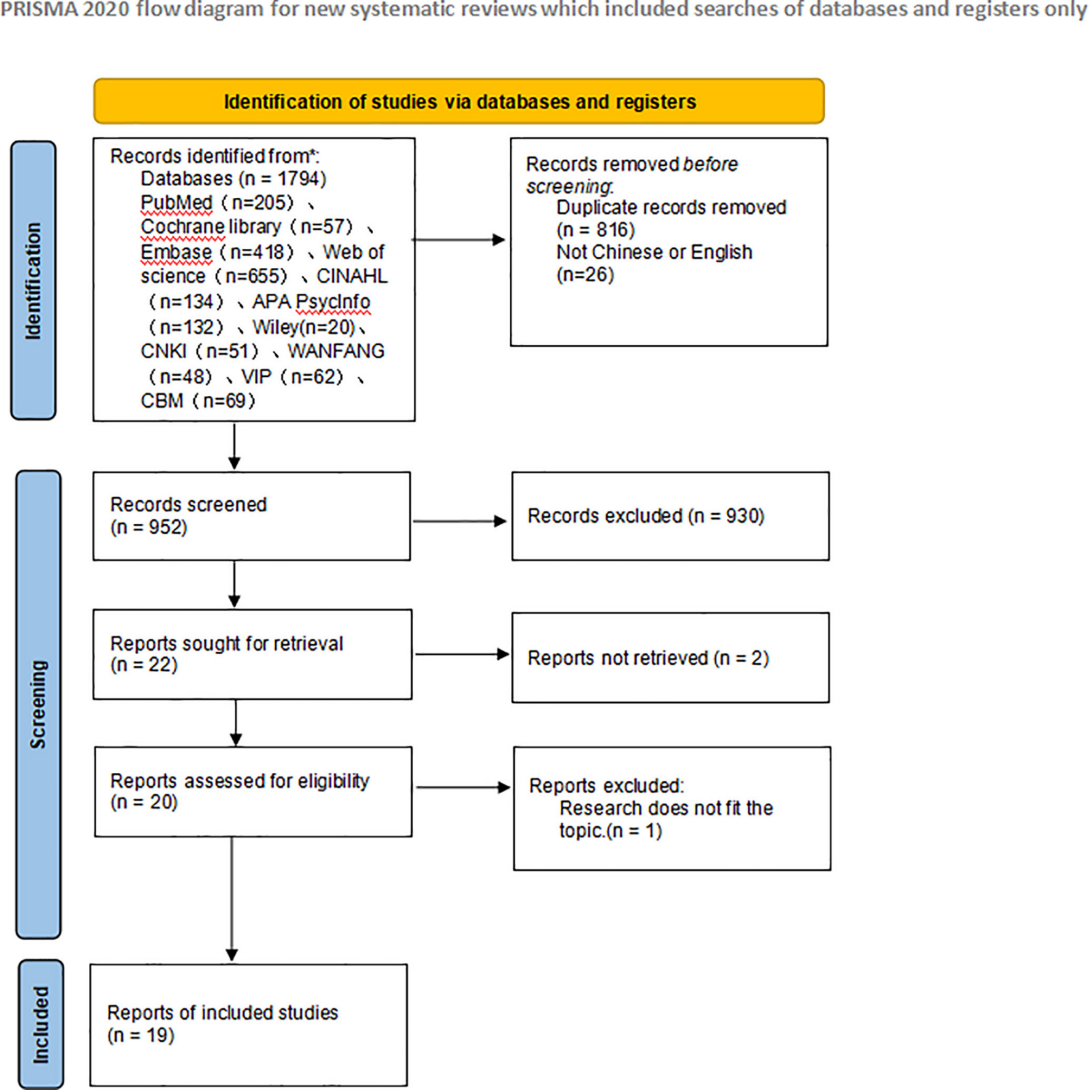
PRISMA flow diagram. CBM = Chinese biomedical literature database, PRISMA = Preferred Reporting Items for Systematic Reviews and Meta-Analyses.

The preliminary search yielded 1794 studies, which was reduced to 952 after the removal of 816 duplicates and the exclusion of 26 studies not in English or Chinese. After a thorough examination of the titles and abstracts, 930 studies were excluded, leaving 22 reports for retrieval. Of these, 2 reports were not retrieved, and 20 full-text articles were assessed for eligibility. After a full-text review, 1 study was excluded because it did not align with the research topic. The final list comprised 19 documents: 10 descriptive qualitative studies, 6 phenomenological studies, 2 interpretive phenomenological studies, and 1 study employing other qualitative methods. The literature screening process is presented in Figure 1.

### 3.2. Methodological quality

The quality evaluations of the 19 included studies comprised 1 Grade A and 18 Grade B studies. The evaluation of the quality of each study is presented in Table 2.

### 3.3. Meta-synthesis results

Through repeated reading, comprehension, and analysis of the 19 included studies, 40 distinct findings (i.e., key statements, themes, or concepts as reported by the authors of the primary studies) were extracted. By synthesizing and integrating these findings, 4 integration themes and 12 subthemes were ultimately derived (see Table 3).

**Table 3:** Integration themes, subthemes, and original descriptive themes.

Main theme	Subtheme	Original descriptive theme
1. Physical changes and how they affect self-image	1.1. Bodily changes after treatment	Body changes caused by surgery: scar impact, breast asymmetry, arm lymphedema, weight gain, etc
		Body changes caused by chemotherapy: hair loss, osteoporosis, joint pain, gastrointestinal complications, etc
		Postoperative lifestyle restrictions
		Fatigue
	1.2. Feeling different and losing feminine identity	Feeling that losing breasts or hair is like losing one’s femininity
		Developing a sense of alienation and estrangement from one’s body
		Unable to accept one’s own physical imperfection
	1.3. Avoiding the mirror because it hurts to look	Feeling distressed upon seeing the incomplete body in the mirror
		Scars in the mirror constantly remind one of being different
		Avoiding looking in the mirror
2. Dealing with others’ judgments and social challenges	2.1. Stress from being seen or exposed	Feeling scrutinized and discriminated against
		Feeling uncomfortable in settings requiring bodily exposure
		Experiencing stress in parent-child interactions due to bodily exposure
		Facing daily challenges with clothing choices
	2.2. Hiding or changing appearance	Using prosthetic breasts, bras, scarves, etc, to alter body shape
		Using wigs, hats, shaved heads, etc, to alter facial appearance
		Changing personal style of dress
	2.3. Staying away from social situations	Refusing to attend public events
		Avoiding leaving home unless necessary, reducing social participation
3. Rebuilding a positive self-image	3.1. Accepting and adjusting to the changed body	Believing feminine qualities remain unchanged
		Realizing breasts do not define womanhood
		Recognizing the body need not conform to external standards
	3.2. Rejecting society’s beauty standards	Not concealing changes to breasts or hair
		Viewing scars as badges of triumph
		Appreciating, listening to, and honestly confronting one’s body
		Transforming body image through exercise and dietary changes
		Maintaining a positive mindset
	3.3. Focusing on what matters most in life	Prioritizing life over appearance
		Cherishing and savoring life more deeply
		Becoming more cheerful and open-minded
4. Body image problems and intimate relationships	4.1. Feeling less attractive and worrying about partners	Medication-induced decreased libido
		Perceived decline in sexual attractiveness
		Sensitivity to partner’s comments about one’s body
		Worries husband won’t accept her appearance
	4.2. Avoiding sex and fearing betrayal	Becomes cautious during sexual encounters
		Avoids dating and couple-only events
		Fears partner will seek other women
	4.3. Ending or limiting marriage because of body image	Decides to divorce after diagnosis
		Chooses not to marry again
		Accepts partner’s infidelity to maintain a superficial marriage

#### 3.3.1. Main theme 1: physical changes and how they affect self-image

##### 3.3.1.1. Subtheme 1: bodily changes after treatment

Body changes in patients with BC are primarily caused by treatment methods. After surgery, patients have numerous scars (“I have a lot of scars”^[18]^) and are highly self-conscious about them (“You see the scar and you know the breast is never the same”^[19]^). Additionally, breast asymmetry occurs (“My breasts are not proportional. There was a visible difference…,”^[20]^ “I felt lopsided, so strange with one boob hanging,”^[21]^ “I have one that is really high and small, while the remaining one is large and droopy”^[22]^), arm lymphedema (“My hand swelled up, more than other arms”^[23]^). Chemotherapy leads to various physical complications, such as hair loss (“Lost all my hair and eyelashes”^[24]^), joint pain (“Bone pain, can’t even straighten my hands,”^[25]^ “I long for the pain in my hands, knees, and back to disappear”^[19]^), decreased gastrointestinal function (“I vomited everything I ate”^[25]^), and menopause (“I went through menopause”^[19]^). Hormone medications cause weight gain (“I gained nearly 40 pounds,”^[26]^ “Suddenly, you don’t wear what you want or what you bought”^[14]^), limitations in daily life (“I can’t even walk down the hall,”^[27]^ “There are many things I can’t do”^[28]^), and fatigue (“I was very tired from so much hospital,”^[23]^ “I don’t want to do anything”^[29]^).

##### 3.3.1.2. Subtheme 2: feeling different and losing feminine identity

Due to the symbolic significance of breasts and hair to females, patients with BC become alienated from and dissatisfied with their own bodies. Patients feel that losing their breasts or hair is akin to losing their feminine identity (“I was glad that I didn’t lose the breast,”^[14]^ “Breasts are so much a part of my life as a woman,”^[20]^ “I felt different from other women… My body had totally changed,”^[30]^ “Breasts represent femininity, but my breast is not there,”^[31]^ “I used to have big hair, but it’s gone now,”^[27]^ “Without hair, I look like a boy now”^[26]^). Patients have a deep disconnect with their bodies (“I do not have a good relationship with my body,”^[18]^ “I don’t recognize my body now,”^[18]^ “My body isn’t mine anymore”^[26]^), and an inability to accept their own physical imperfections (“I’m dissatisfied with my arms and breasts,”^[23]^ “I don’t like my body,”^[18]^ “I’d only give myself a 2 out of 10 – I dislike every part of myself,”^[29]^ “I felt ashamed whenever I went outside of the house”^[28]^).

##### 3.3.1.3. Subtheme 3: avoiding the mirror because it hurts to look

Gazing at their fragmented bodies and distinctive scars in the mirror triggers traumatic memories and evokes avoidance behaviors in patients with BC. Patients see their incomplete body in the mirror, causing deep distress (“The sight of my naked body disturbs me, because I am forced to rethink the lived experience,”^[18]^ “Seeing my nearly bald head terrifies me,”^[24]^ “I can’t even count how many times I’ve cried in front of the mirror,”^[26]^ “You’re not the same as all other women,”^[14]^ “When I went into the bathroom and looked in the mirror, I felt so bad”^[31]^). The scars in the mirror constantly remind patients of their distinctiveness (“When I look at myself in the mirror, it is as if I saw the body of another woman,”^[18]^ “The scar constantly reminds me I had cancer,”^[26]^ “It just was not me. It was someone completely foreign, standing there at that moment”^[14]^), leading BC survivors to avoid looking in the mirror (“I avoid looking in the mirror as much as possible,”^[18]^ “I only look in the mirror when fully dressed,”^[26]^ “I no longer look in the mirror at all”^[32]^).

#### 3.3.2. Main theme 2: dealing with others’ judgments and social challenges

##### 3.3.2.1. Subtheme 4: stress from being seen or exposed

The bodily exposure dilemmas of BC patients generally occur in daily social interactions, parent-child relationships, and clothing choices, where they face stigmatization pressures under societal scrutiny. BC patients are exposed to societal judgment and discrimination (“I was a freak show for the whole village,”^[33]^ “They always stare at me,”^[26]^ “I sometimes get denounced by outsiders that I had the mastectomy,”^[28]^ “They comment on my body scars”^[20]^). These experiences deeply troubled the patients (“I felt that I was worthless and I was really bothered by the questioning and comments of other people”^[30]^), making them uncomfortable in settings requiring bodily exposure (“At the pool, I had to change privately. I don’t need everyone to know that I have this problem,”^[14]^ “I’m extra careful when changing clothes”^[26]^), causing them to experience pressure in parent-child interactions (“I feel embarrassed in front of my son,”^[14]^ “My kids can’t accept how I look”^[32]^), and to face daily dressing dilemmas (“I spend a lot of time choosing the appropriate bras,”^[34]^ “how would I wear the summer clothes?,”^[28]^ “I haven’t taken off my shirt for 10 years”^[14]^). Patients pin their hopes on reconstructive surgery to resolve all dressing-related issues (“I was glad my surgical team informed me of the possibility of reconstruction,”^[22]^ “No more worrying about prostheses or bra combinations”^[26]^).

##### 3.3.2.2. Subtheme 5: hiding or changing appearance

Patients with BC reshape their body image by concealing and modifying their physical appearance to meet societal expectations. Patients alter their appearance through prostheses, bras, scarves, and other means (“Wearing a prosthesis makes me look normal,”^[26]^ “I cover my chest with a scarf or something else,”^[20]^ “I sleep with my bra on”^[35]^), or alter their appearance through wigs, hats, or shaving their heads (“I definitely wore a wig or a scarf or something like that because you really look completely different,”^[14]^ “I always wear a hat when going out,”^[25]^ “I wore a wig that looked just like my real hair, so my mother didn’t realize I was sick”^[24]^), or altering clothing styles (“Earlier, I preferred to wear slim-fitting dresses, but now I wear loose-fitting clothes to avoid people’s glances,”^[31]^ “I don’t wear sleeveless tops anymore,”^[26]^ “I choose lace-trimmed camisoles to cover scars”^[25]^).

##### 3.3.2.3. Subtheme 6: staying away from social situations

Patients with BC exhibit social withdrawal due to perceived bodily shame. For instance, patients may refuse to attend public events (“my body image held me back,”^[20]^ “I shouldn’t go to social gatherings or weddings,”^[31]^ “The thought of undressing makes me absolutely avoid swimming pools”^[26]^), avoid leaving home unless necessary and reduce social participation (“I don’t want to go out, I just want to stay home,”^[29]^ “I lock myself indoors watching TV,”^[29]^ “When I see someone I know ahead, I quickly hide,”^[24]^ “I refused to visit anyone to avoid seeing their pity”^[30]^).

#### 3.3.3. Main theme 3: rebuilding a positive self-image

##### 3.3.3.1. Subtheme 7: accepting and adjusting to the changed body

Patients with BC gradually embrace themselves, transform scars into badges of life, and assert control over their bodies wherever possible. Some no longer conceal changes to their breasts or hair (“I accepted shaving my head – if I’m sick, I’m sick,”^[25]^ “I don’t need to hide or erase them”^[26]^), viewing scars as badges of victory (“I wear them like battle scars and am proud of them”^[20]^). Patients begin to appreciate, listen to, and confront their bodies honestly (“I talk to my body often and ask it to support me, not to fight me that we are one,”^[18]^ “Tell myself I look great”^[26]^) and transform their body image through exercise and dietary awareness (“I started working out,”^[26]^ “maintaining a balanced diet,”^[20]^ “consuming healthy foods”^[20]^), while cultivating a positive mindset (“meditation helps me find inner peace and focus”^[26]^).

##### 3.3.3.2. Subtheme 8: rejecting society’s beauty standards

At the same time as self-acceptance, BC patients deconstruct the symbolic meanings given to their breasts by society and refuse to accept external pressures imposed by social and cultural norms. Patients claim that femininity remains unchanged after mastectomy (“My appearance may have changed, but my feminine qualities haven’t,”^[26]^ “My femininity doesn’t depend on breasts”^[26]^). They recognize that breasts do not define womanhood (“Breasts don’t define me as a woman,”^[19]^ “Losing a breast hasn’t stopped me from doing anything – this is just me with one breast”^[19]^, “Losing breasts doesn’t determine whether you’re a woman”^[29]^), and affirm that their bodies need not conform to external standards (“It is beautiful in itself”^[20]^).

##### 3.3.3.3. Subtheme 9: focusing on what matters most in life

After undergoing rehabilitation, BC patients begin to reorder their life values, prioritize life over an obsession with physical perfection, and reshape a more open-minded attitude toward life. Patients who have regained their health believe that life is more important than appearance (“As long as I’m healthy, breasts don’t mean a lot,”^[31]^ “No breasts are not the most important thing in the world,”^[14]^ “I won’t waste another second”^[19]^). They cherish and savor life more deeply (“Life is certainly more important than appearance,”^[34]^ “I’m grateful my body recovered and allowed me to have a child,”^[18]^ “I remind myself to enjoy the present moment”^[19]^), and become more cheerful and open-minded (“I’m more cheerful and talkative than before,”^[25]^ “I believe I’m living at the most perfect moment”^[34]^).

#### 3.3.4. Main theme 4: body image problems and intimate relationships

##### 3.3.4.1. Subtheme 10: feeling less attractive and worrying about partners

In intimate relationships, BC treatment triggers diminished libido and cognitive dissonance regarding one’s sexual attractiveness, plunging patients into dual anxieties regarding partner loyalty and marital stability. Medications cause reduced sexual desire (“During chemo, I had absolutely no interest in sex,”^[26]^ “I rarely feel that need,”^[24]^ “The drugs put me into menopause”^[24]^). Patients perceive a decline in their own sexual attractiveness (“I’ve lost all confidence in my naked body,”^[22]^ “I wasn’t married, and I doubt anyone would find me attractive”^[27]^). They become preoccupied with their partner’s reactions to their body (“He never touches my scar or my new breast,”^[26]^ “His avoidance of touching the scar hurts me”^[20]^), and fear that their husbands would reject their appearance (“After 8 years of marriage, I still refuse to be fully naked in front of my husband,”^[19]^ “I wore a scarf all the time, especially when it came to my husband seeing me without breasts, hair, and eyebrows, and I thought he might leave me and marry someone else”^[30]^).

##### 3.3.4.2. Subtheme 11: avoiding sex and fearing betrayal

BC patients’ avoidance of sexual contact and persistent anxiety about partner fidelity trigger a crisis of trust in rebuilding intimate relationships. Patients become cautious during sexual encounters (“I’m wary when my husband touches my breasts,”^[23]^ “I only have sex after consulting my doctor, and very rarely,”^[32]^ “I used to refuse sex out of fear of recurrence, and still don’t have it often,”^[25]^ “Even though the doctor said it’s okay, I’m unwilling”^[25]^), refuse or avoid dating, and steer clear of couple-only events (“For now, I’ve decided not to date or have sex with anyone,”^[19]^ “I worry about recurrence and consistently avoid serious relationships,”^[19]^ “I avoid events requiring joint participation”^[31]^), and fear that partners may seek other women (“I fear my husband will leave me for another woman”^[30]^).

##### 3.3.4.3. Subtheme 12: ending or limiting marriage because of body image

BC patients experience self-denial stemming from body image issues, leading them to proactively dissolve marriages, abandon the prospects of remarriage, and tacitly permit their partners’ infidelity through financial compensation. After diagnosis, some decide to divorce (“I decided to end this hollow 18-year marriage,”^[19]^ “If I have to undergo a mastectomy, I’ll break up with my fiancé”^[28]^), choose not to enter marriage at all (“I never intended to find another one,”^[24]^ “Who would marry a sick woman with a deformed body and long-term health issues? I no longer think about that”^[28]^), or accept partners’ infidelity to maintain superficial marriages (“I accept his cheating. When he made demands, I said: I’ll give you some money, go find someone else,”^[24]^ “I told him [my husband] I don’t mind how much he spends outside, as long as he comes back clean”^[24]^).

## 4. Discussion

The integrated findings revealed that physical defects in patients with BC can trigger mind-body mutual exclusion, and mirror trauma further exacerbates body image cognitive dissonance. Early identification and intervention are crucial for mitigating body image disturbances in this population. The Body Image Scale is recommended for measuring the level of body image disturbances in BC patients due to its well-established psychometric properties and widespread use.^[36]^ Previous studies have shown that exercise therapy, acceptance and commitment therapy, cognitive behavioral therapy, nurse-led case intervention, and information and emotional counseling interventions can effectively reduce body image disturbances in BC patients.^[37]^ However, research on interventions specifically targeting body image mirror trauma in BC patients remains limited. Mirror exposure therapy instructs patients to systematically observe and describe their own bodies, relieve pain, and enhance acceptance.^[38]^ While it has been shown to be effective for the body image of patients with eating disorders, its application in BC care is underexplored. Therefore, future studies should investigate the applicability and efficacy of mirror therapy in this population.

Our synthesis indicates that negative body image traps patients in bodily stigmatization, leading to social withdrawal and sexual intimacy issues. We propose establishing a 4-level social support network covering families, communities, organizations, and medical systems to help patients rebuild their self-awareness and overcome adversity. A cooperative care plan can enhance the sense of mutual support among family members and make BC patients feel a sense of self-worth, such as light household chores, joint rehabilitation exercises, and collaborative planning of treatment responsibilities and home care routines.^[39]^ The community should strengthen the publicity of government health policies, such as the “two cancers” rescue plan, to protect health rights and interests.^[40]^ Nonprofit international organizations can run positive body image campaigns to restore patients’ confidence and facilitate reintegration programs for economic independence.^[41]^ To address negative body image associated with BC, medical institutions can develop educational materials to eliminate societal stigma and showcase postoperative scar management and prosthetic breast use to enhance body confidence. Peer support programs should be vigorously promoted, and patients should be organized to share their experiences in rebuilding their physical confidence.^[42]^ This helps newly diagnosed BC patients to view scars, breast loss, and arm lymphedema from diverse perspectives, thus mitigating societal stigma.

The integrated findings suggest that marital crises among patients with BC stem from self-deprecation and withdrawal from intimacy, triggered by impaired body image, necessitating the reconstruction of partner interaction patterns and trust-based relationships. Research demonstrates that effective communication, serving as the foundation for joint coping, increases spousal trust and improves family functioning.^[43]^ The “Supporting Her Recovery” program and the “Healthy Family Function Management” program can develop practical communication skills.^[44]^ Adapted to local contexts, these programs can form the basis for personalized interventions. Medical service providers should guide couples to frankly discuss physical changes and redefine scars as “badges of victory,” thus enhancing patients’ confidence in intimate relationships. Encouraging partners to provide positive physical touch and verbal compliments can clearly reflect an accepting attitude and build trust. This process can turn the marriage crisis into an opportunity for common growth and help BC patients change from “fear of being watched” to “feeling cherished.”

## 5. Limitations

Of the 19 papers included in the study, 14 failed to consider the influence of the researchers’ own values and cultural backgrounds. The psychological experiences and needs of people with body image issues are influenced by regional culture, which may lead to differences in the results. Studies were selected based on the inclusion criteria of the literature review. Only qualitative studies published in English- or Chinese-indexed journals were included. It is acknowledged that the search process may have omitted certain gray literature sources, a potential factor that could have led to the incorporation of biased information.

## 6. Conclusion

This study systematically integrates the complex psychological and social adaptation processes experienced by patients with BC when confronted with changes in body image, emphasizing the importance of early identification and intervention. Future research should explore effective intervention measures targeting BC body image, construct socially embodied support networks, and rebuild marital and trust relationships to promote comprehensive recovery among BC patients. Although this study incorporates findings from multiple qualitative research papers, it is subject to certain limitations, as qualitative research inevitably involves subjective biases. Future longitudinal studies could further examine the psychological trajectory of BC body image experiences, develop and validate intervention protocols, and help patients overcome body-related challenges while enhancing their self-acceptance and tolerance.

## Acknowledgments

The authors gratefully acknowledge the original research reports included in this study.

## Author contributions

**Conceptualization:** Long Yao, Li Ling.

**Data curation:** Long Yao, Li Ling, Tuo Xuemei, Yu Dan.

**Validation:** Long Yao.

**Formal analysis:** Tuo Xuemei.

**Methodology:** Tuo Xuemei, Yu Dan.

**Software:** Yu Dan.

**Supervision:** Huang Yali.

**Writing – original draft:** Long Yao.

**Writing – review & editing:** Huang Yali.


