# Supplementary material for: A meta-synthesis of qualitative research on negative body image among female breast cancer patients: Perceptions, stigma, and identity negotiation
Source: Medicine (Baltimore). 2026 Jul 3;105(27):e49592. doi: 10.1097/MD.0000000000049592 (PMC13337039; doi:10.1097/MD.0000000000049592)
Supplement: Supplementary file 1 [file medi-105-e49592-s001.docx]

Appendix 1: Search Strategy

| **PubMed**  #1 "breast neoplasms"[MeSH Terms] OR "breast cancer"[Title/Abstract] OR "breast tumor*"[Title/Abstract]  #2 “body image”[MeSH Terms] OR“body images”[Title/Abstract] OR “body identity”[Title/Abstract] OR “identity, body”[Title/Abstract] OR “body representation”[Title/Abstract] OR “image, body”[Title/Abstract] OR “body representations”[Title/Abstract] OR “Representation, Body”[Title/Abstract] OR “body schema”[Title/Abstract] OR “body schemas”[Title/Abstract] OR “schema, body”[Title/Abstract]  #3 “qualitative research”[MeSH Terms] OR “qualitative research”[Title/Abstract] OR “qualitative method”[Title/Abstract] OR “qualitative study”[Title/Abstract] OR “phenomenolog*”[Title/Abstract] OR “lived experience*”[Title/Abstract] OR “grounded theory” [Title/Abstract] OR “ethnograph*” [Title/Abstract] OR “case study” [Title/Abstract] OR “action research” [Title/Abstract] OR “discourse analy*” [Title/Abstract] OR “interview*” [Title/Abstract] OR “focus group*” [Title/Abstract] OR “participant observ*” [Title/Abstract] OR “field note*” [Title/Abstract] OR “content analy*”[Title/Abstract] OR “thematic analy*”[Title/Abstract] OR “Colaizzi”[Title/Abstract] OR “constant comparison” [Title/Abstract] OR “constant comparative analysis”[Title/Abstract]  #4 #1 AND #2 AND #3 | 205 |
| --- | --- |
| **Wiley**  "breast neoplasms" OR "breast cancer" OR "breast tumor*"  “body image” OR “body images” OR “body identity” OR “identity, body” OR “body representation” OR “image, body” OR “body representations” OR “Representation, Body” OR “body schema” OR “body schemas” OR “schema, body”  “qualitative research” OR “qualitative research” OR “qualitative method” OR “qualitative study” OR “phenomenolog*” OR “lived experience*” OR “grounded theory” OR “ethnograph*” OR “case study” OR “action research” OR “discourse analy*” OR “interview*” OR “focus group*” OR “participant observ*” OR “field note*” OR “content analy*” OR “thematic analy*” OR “Colaizzi” OR “constant comparison” OR “constant comparative analysis” | Title 1  Ab 19 |
| **APA PsycInfo**  3 Results for Title: "breast neoplasms" OR Title: "breast cancer" OR Title: "breast tumor*" AND Title: “body image” OR “body images” OR “body identity” OR “identity, body” OR “body representation” OR “image, body” OR “body representations” OR “Representation, Body” OR “body schema” OR “body schemas” OR “schema, body” AND Title: “qualitative research” OR “qualitative research” OR “qualitative method” OR “qualitative study” OR “phenomenolog*” OR “lived experience*” OR “grounded theory” OR “ethnograph*” OR “case study” OR “action research” OR “discourse analy*” OR “interview*” OR “focus group*” OR “participant observ*” OR “field note*” OR “content analy*” OR “thematic analy*” OR “Colaizzi” OR “constant comparison” OR “constant comparative analysis  129 Results for Abstract: "breast neoplasms" OR Abstract: "breast cancer" OR Abstract: "breast tumor*" AND Abstract: “body image” OR “body images” OR “body identity” OR “identity, body” OR “body representation” OR “image, body” OR “body representations” OR “Representation, Body” OR “body schema” OR “body schemas” OR “schema, body” AND Abstract: “qualitative research” OR “qualitative research” OR “qualitative method” OR “qualitative study” OR “phenomenolog*” OR “lived experience*” OR “grounded theory” OR “ethnograph*” OR “case study” OR “action research” OR “discourse analy*” OR “interview*” OR “focus group*” OR “participant observ*” OR “field note*” OR “content analy*” OR “thematic analy*” OR “Colaizzi” OR “constant comparison” OR “constant comparative analysis” | TI 3  AB 129 |
| **CINAHL Complete (EBSCO Host)**  XB ("breast neoplasms" OR "breast cancer" OR "breast tumor*") AND XB (“body image” OR “body images” OR “body identity” OR “identity, body” OR “body representation” OR “image, body” OR “body representations” OR “Representation, Body” OR “body schema” OR “body schemas” OR “schema, body”) AND XB (“qualitative research” OR “qualitative research” OR “qualitative method” OR “qualitative study” OR “phenomenolog*” OR “lived experience*” OR “grounded theory” OR “ethnograph*” OR “case study” OR “action research” OR “discourse analy*” OR “interview*” OR “focus group*” OR “participant observ*” OR “field note*” OR “content analy*” OR “thematic analy*” OR “Colaizzi” OR “constant comparison” OR “constant comparative analysis”)  Note:Search titles and abstracts, limited to English and Chinese | 134 |
| **WOS√**  ((TS=("breast neoplasms" OR "breast cancer" OR "breast tumor*")) AND TS=(“body image” OR “body images” OR “body identity” OR “identity, body” OR “body representation” OR “image, body” OR “body representations” OR “Representation, Body” OR “body schema” OR “body schemas” OR “schema, body”)) AND TS=(“qualitative research” OR “qualitative research” OR “qualitative method” OR “qualitative study” OR “phenomenolog*” OR “lived experience*” OR “grounded theory” OR “ethnograph*” OR “case study” OR “action research” OR “discourse analy*” OR “interview*” OR “focus group*” OR “participant observ*” OR “field note*” OR “content analy*” OR “thematic analy*” OR “Colaizzi” OR “constant comparison” OR “constant comparative analysis”)  Note:TOPIC，limited to English and Chinese | 655 |
| **EMBASE**  Quick search  TI/AB  ('breast neoplasms':ab,ti OR 'breast cancer':ab,ti OR 'breast tumor*':ab,ti) AND ('body image':ab,ti OR 'body images':ab,ti OR 'body identity':ab,ti OR 'identity, body':ab,ti OR 'body representation':ab,ti OR 'image, body':ab,ti OR 'body representations':ab,ti OR 'representation, body':ab,ti OR 'body schema':ab,ti OR 'body schemas':ab,ti OR 'schema, body':ab,ti) AND ('qualitative research':ab,ti OR 'qualitative method':ab,ti OR 'qualitative study':ab,ti OR 'phenomenolog*':ab,ti OR 'lived experience*':ab,ti OR 'grounded theory':ab,ti OR 'ethnograph*':ab,ti OR 'case study':ab,ti OR 'action research':ab,ti OR 'discourse analy*':ab,ti OR 'interview*':ab,ti OR 'focus group*':ab,ti OR 'participant observ*':ab,ti OR 'field note*':ab,ti OR 'content analy*':ab,ti OR 'thematic analy*':ab,ti OR 'colaizzi':ab,ti OR 'constant comparison':ab,ti OR 'constant comparative analysis':ab,ti) | 418 |
| **CNKI**  (TKA=乳腺癌 OR TKA=乳癌 OR TKA=乳腺肿瘤 OR TKA=乳房肿瘤) AND (TKA=身体意象 OR TKA=身体形象 OR TKA=身体心像 OR TKA=体像 OR TKA=身体自我 OR TKA=外表改变 OR TKA=自我形象) AND (TKA=感受 OR TKA=体验 OR TKA=经历 OR TKA=质性研究 OR TKA=定性研究 OR TKA=现象学) | 51 |
| **WANFANG**（只检索期刊、学位论文）  专业检索：  (“乳腺癌” OR “乳癌” OR “乳腺肿瘤” OR “乳房肿瘤”) AND (“身体意象” OR “身体形象” OR “身体心像” OR “体像” OR “身体自我” OR “外表改变” OR “自我形象”) AND (“感受” OR “体验” OR “经历” OR “质性研究” OR “定性研究” OR “现象学”) | 48 |
| **VIP**  主题检索，精确，只检索期刊和学位论文，同义词扩展，中英文扩展  (“乳腺癌” OR “乳癌” OR “乳腺肿瘤” OR “乳房肿瘤”) AND (“身体意象” OR “身体形象” OR “身体心像” OR “体像” OR “身体自我” OR “外表改变” OR “自我形象”) AND (“感受” OR “体验” OR “经历” OR “质性研究” OR “定性研究” OR “现象学”) | 62 |
| **CBM**  ( "乳腺癌"[中文标题:智能] OR "乳癌"[中文标题:智能] OR "乳腺肿瘤"[中文标题:智能] OR "乳房肿瘤"[中文标题:智能]) AND( "身体意象"[中文标题:智能] OR "身体形象"[中文标题:智能] OR "身体心像"[中文标题:智能] OR "体像"[中文标题:智能] OR "身体自我"[中文标题:智能] OR "外表改变"[中文标题:智能] OR "自我形象"[中文标题:智能]) AND( "感受"[中文标题:智能] OR "体验"[中文标题:智能] OR "经历"[中文标题:智能] OR "质性研究"[中文标题:智能] OR "定性研究"[中文标题:智能] OR "现象学"[中文标题:智能])  [( "乳腺癌"[摘要:智能] OR "乳癌"[摘要:智能] OR "乳腺肿瘤"[摘要:智能] OR "乳房肿瘤"[摘要:智能]) AND( "身体意象"[摘要:智能] OR "身体形象"[摘要:智能] OR "身体心像"[摘要:智能] OR "体像"[摘要:智能] OR "身体自我"[摘要:智能] OR "外表改变"[摘要:智能] OR "自我形象"[摘要:智能]) AND( "感受"[摘要:智能] OR "体验"[摘要:智能] OR "经历"[摘要:智能] OR "质性研究"[摘要:智能] OR "定性研究"[摘要:智能] OR "现象学"[摘要:智能])](javascript:toDoRelimitSearch();) | TI:3  AB:66 |
|  | 1794 |
